# SERCA is critical to control the Bowditch effect in the heart

**DOI:** 10.1038/s41598-018-30638-9

**Published:** 2018-08-20

**Authors:** Darío Balcazar, Victoria Regge, Manuela Santalla, Heiko Meyer, Achim Paululat, Alicia Mattiazzi, Paola Ferrero

**Affiliations:** 1 Centro de Investigaciones Cardiovasculares – CONICET/Universidad Nacional de la Plata, La Plata, Argentina; 2 Centro de Investigaciones Cardiovasculares – CONICET/Universidad Nacional de la Plata and Departamento de Ciencias Básicas y Experimentales –UNNOBA, La Plata, Argentina; 3 University of Osnabrück, Biology, Department of Zoology and Developmental Biology, Barbarastraße 11, 49076 Osnabrück, Germany

## Abstract

The Bowditch effect or staircase phenomenon is the increment or reduction of contractile force when heart rate increases, defined as either a positive or negative staircase. The healthy and failing human heart both show positive or negative staircase, respectively, but the causes of these distinct cardiac responses are unclear. Different experimental approaches indicate that while the level of Ca^2+^ in the sarcoplasmic reticulum is critical, the molecular mechanisms are unclear. Here, we demonstrate that *Drosophila melanogaster* shows a negative staircase which is associated to a slight but significant frequency-dependent acceleration of relaxation (FDAR) at the highest stimulation frequencies tested. We further showed that the type of staircase is oppositely modified by two distinct SERCA mutations. The dominant conditional mutation SERCA^A617T^ induced positive staircase and arrhythmia, while SERCA^E442K^ accentuated the negative staircase of wild type. At the stimulation frequencies tested, no significant FDAR could be appreciated in mutant flies. The present results provide evidence that two individual mutations directly modify the type of staircase occurring within the heart and suggest an important role of SERCA in regulating the Bowditch effect.

## Introduction

The Bowditch effect or staircase phenomenon is the intrinsic property of the heart to either increase or decrease the force of contraction in response to an increment in pacing rate. It was described by Bowditch in 1871^1^. Indeed, the Bowditch effect is important to determine the cardiovascular response to exercise, constituting a major contributor to the intrinsic myocardial reserve^2^. A positive staircase effect occurs in normal individuals and it is believed that 40% of the increase in cardiac output (volume of blood pumped by the ventricle in one minute) depends on the relationship between strength and frequency^3^. In people with heart failure, the force-frequency relationship is null or inverse (negative staircase) and the heart is unable to meet the blood circulation requirements even under low-stress exercises^4,5^.

The most important mechanisms that underlie the staircase phenomenon and its reversal are associated with Ca^2+^ handling and mishandling in cardiac cells. This involves proteins that participate in the excitation-contraction coupling, such as the sarcoplasmic reticulum (SR) Ca^2+^-ATPase (SERCA). A direct correlation between the reduced levels of SR Ca^2+^ uptake, decreased SERCA expression, -a hallmark of cardiac failure-, and a depressed force-frequency response was reported in the failing myocardium^6^. Furthermore, mutations in SERCA2 account for a dominant skin disease (Darier disease and its clinical variants), with the effects of several of these mutations being exacerbated by elevated ambient temperatures^7^. However, a causal relation between SERCA mutations and impaired cardiac activity, has not been established yet. The mutations SERCA^E442K^ and SERCA^A617T^ were identified in *D*. *melanogaster* genetic screenings and involve amino acids changes in the hinge region of the protein, thus disrupting pumping activity^8–10^. Here, we investigated the physiological impact of these mutations on heart activity in *D*. *melanogaster*, with special emphasis on the staircase phenomenon.

## Results

### *D*. *melanogaster* exhibits negative staircase

**Figure 1:**
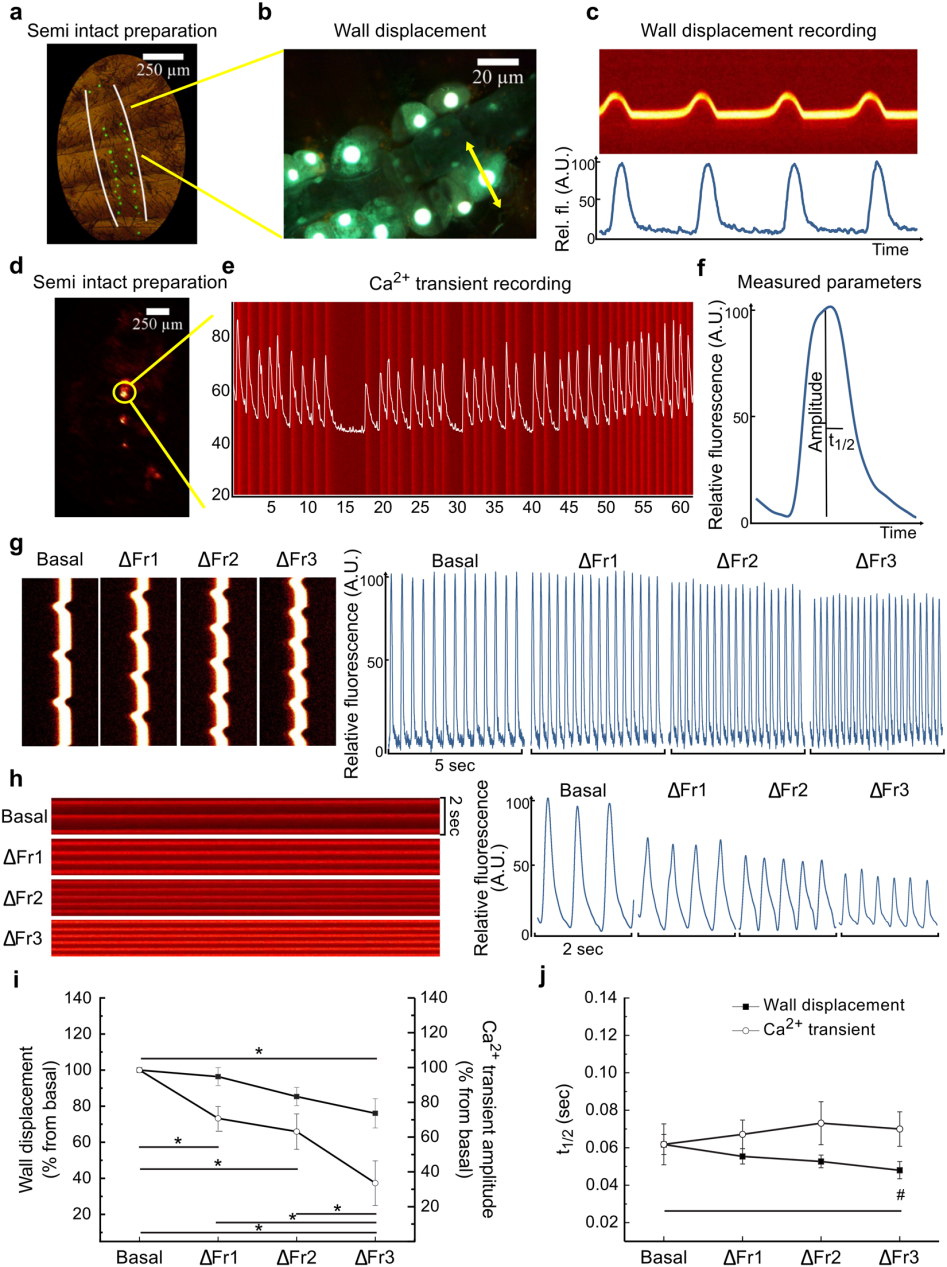
*D*. *melanogaster* exhibits negative staircase. (**a**) Semi intact preparation of a fly harboring the reporter system *hand*C-GFP. (**b**) An amplified fluorescence image showing the fluorescent cardiomyocytes and pericardial cells. The arrow indicates the cell displacement trajectory. (**c**) Raw (above) and digitized (below) cell movements. (**d**) Semi intact preparation of a fly harboring the reporter system GCaMP3. (**e)** Images of intracellular Ca^2+^ transients tracked in the conical chamber. The recording represents an arrhythmic heart. (**f**) Amplified Ca^2+^ transient to indicate the different parameters analyzed. (**g)** Representative recording of mechanical activity showing lateral displacements of a single cell after three successive 0.5 Hz increments in stimulation frequency (ΔFr1, ΔFr2, ΔFr3) from basal (B) spontaneous heart rate. Right panel: twitches obtained after digitalization of images using an algorithm based in phyton language. (**h**) Left panel: representative line scans of calcium transient after three successive 0.5 Hz increments in stimulation frequency. Right panel: Digitized images of the respective line scans. (**i)** Quantification of mechanical activity (black symbols) and Ca^2+^ transients (white symbols), reveals the negative staircase in *D*. *melanogaster*. N = 20, 19, 13, 5 for mechanical activity data at basal, ΔFr1, ΔFr2, and Δ Fr3 respectively. N = 10, 10, 7, 4 for Ca^2+^ transients data at basal, ΔFr1, ΔFr2, and ΔFr3 respectively. (**j**) *D*. *melanogaster* exhibit significant FDAR only at the highest frequency. Quantification of half relaxation time of cell movement and Ca^2+^ transients. N = 11, 10, 5, 4 for mechanical activity data at basal, ΔFr1, ΔFr2, and ΔFr3 respectively. N = 9, 9, 8, 4 for Ca^2+^ transients data at basal, ΔFr1, ΔFr2, and ΔFr3 respectively. Two way ANOVA followed by Tukey’s test for multiple comparisons was used for statistical analysis.*, ^*#*^P < 0.05.

We first characterized staircase in adult *D*. *melanogaster*, by analyzing heart movements and cytosolic Ca^2+^ levels during each contraction cycle (Ca^2+^ transients). We used semi intact preparations (Fig. 1a) and measured cardiac wall movements in 7–11 days old flies harboring the reporter *hand*C-GFP, expressed in cardiomyocytes and pericardial cells (Supplementary Movie 1)^11^. The movement of either the GFP-fluorescent cardiomyocytes or the associated pericardial cells was then traced (Fig. 1b) to obtain the raw and digitized data as shown in Fig. 1c. Intracellular Ca^2+^ transient recordings (Fig. 1e,f) were obtained by using the Ca^2+^ sensor GCaMP3, which monitored increments of Ca^2+^ in the cytosol preceding each contraction (Fig. 1d)^12^.

To determine the type of staircase in *D*. *melanogaster*, fly hearts were subjected to stepwise increases in stimulation frequency. Figure 1g,h show the typical responses of mechanical activity and Ca^2+^ transients, respectively, to three successive increments in stimulation frequency above basal spontaneous frequency. Figure 1i shows that the Ca^2+^ transient amplitude displays a significant reduction with the increment of stimulation frequency while reduction of the wall displacement is significantly less at each frequency applied. Supplementary Figure S1 shows that the fluorescent signal is preserved over time for Ca^2+^ transient recordings. Therefore, the results reflect the physiological effects of incrementing the frequency of stimulation on Ca^2+^ handling. Supplementary Figure S2 indicates that unlike what is observed in other species^2^, diastolic Ca^2+^ does not change with an increment in frequency. Results presented in Fig. 1i indicated a negative staircase in *D*. *melanogaster* wild type (CONTROL) adults. Thus, the *Drosophila* heart behaves similar to what has been determined for small mammals, such as rats and mice, following a change in stimulation frequency^13^.

### The frequency-dependent acceleration of relaxation (FDAR) is small and only occurs at the highest stimulation frequencies explored

Independently of the nature of the staircase, an increment in the frequency of stimulation induced an acceleration of relaxation (FDAR)^13^. We measured the time to half of relaxation (t½), as an index of relaxation of both, twitch shortening and Ca^2+^ transients. Increasing stimulation frequency produced a minimal reduction in t½ of twitch shortening that only reached statistical significance at the highest stimulation frequency explored. This decrease was completely absent in the corresponding Ca^2+^ transient (Fig. 1j). The mechanism of FDAR is not clear yet but in mammals appears to be linked to the activity of Ca^2+^-calmodulin-dependent protein kinase II (CaMKII)^14–16^. Human homologous CaMKII is present in *D*. *melanogaster*^17^, and corresponding targets of phosphorylation should be study to understand the modest FDAR observed in twitch shortening in this model associated with the absence of FDAR in intracellular Ca^2+^ transients.

### SERCA mutations in *D*. *melanogaster* change the CONTROL pattern of staircase

SERCA is responsible for the SR Ca^2+^ reuptake and is the main contributor to cardiac relaxation. Human SERCA2 and *Drosophila* SERCA (dSERCA) share 73% identity at the amino acid level and 96% of the 89 sites altered by mutations are conserved in humans and *Drosophila* (see Supplementary Fig. S3)^8,18^. A positive staircase of the healthy heart is mainly dependent on the SR Ca^2+^ load, whereas a negative staircase in humans is a hallmark of heart failure associated with a diminished expression of SERCA2a^6^. Moreover, mice models exhibiting reduced SERCA expression are characterized by a negative staircase compared to animals with unaltered levels of this protein^19^. Our results in *D*. *melanogaster* show that the negative staircase represents the wild type situation. In a new series of experiments we explored whether two different SERCA mutations modify the staircase pattern observed in wild type *D*. *melanogaster*.

We analyzed two different conditional and dominant mutations that cause global disruption of SERCA activity. SERCA^A617T^ replaces alanine 617 for threonine^10^ and causes a temperature sensitive-incoordination phenotype. This mutation has been proposed to result in improper coupling of the ATPase cycle and impared gating of the transmembrane domain. Elevated temperatures facilitate a shift to this malfunctioning state in PAII (Type II P-type ATPases) family pumps that are affected by dominant mutations characterized in non-cardiac pathologies^10^. On the other hand, SERCA^E442K^ elicits a dominant phenotype of paralysis induced by heat shock, thus causing recessive lethality. Furthermore, SERCA^E442K^ changes glutamic acid 442 to lysine^8,9^. This mutation has been proposed to influence the binding of ATP and the resulting conformational change of the protein. Corresponding flies exhibit paralysis induced by exposure of individuals at a restrictive temperature of 41 °C^8^. Presence of the E442K mutation has been reported to reduce heart rate in larvae, pupae and intact adult flies after heat shock, along with enlarged cardiac dimensions in adult hearts^9,20^.

**Figure 2:**
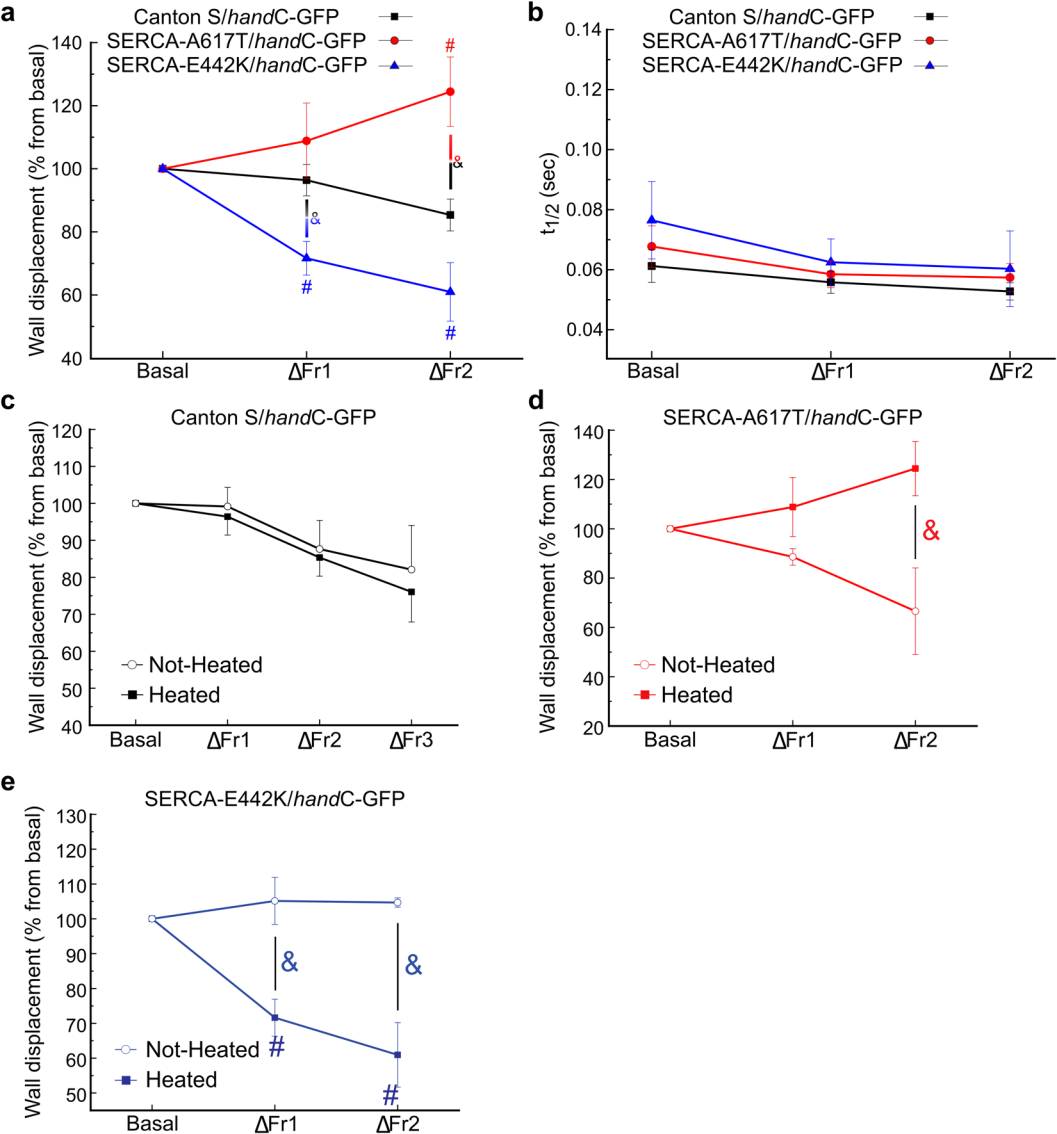
SERCA mutations modify the staircase pattern in *Drosophila melanogaster*. (**a**) Mutant SERCA^A617T^ animals exhibit positive staircase and mutant SERCA^E442K^ animals negative staircase, compared to control individuals. (**b)** The time of half to relaxation did not change with an increment stimulation frequency in all transgenic lines. Amplitude and t_1/2_: Control: N = 20, 1, 9. SERCA^A617T^ N = 8, 10, 8. SERCA^E442K^ N = 6, 5, 4. Two way ANOVA followed by Tukey’s test for multiple comparisons was used for statistical analysis. *P < 0.05 (between strains only in basal stimulation), ^#^P < 0.05 (between strains in different frequency stimulation), & P < 0.05 (between strains). (**c**–**e)** A subgroup of flies were previously exposed (or not) to heat shock (closed and open symbols, respectively). The results are expressed as percentage of change related to the basal cardiac frequency. Data represent mean values ± S.E., and significance was calculated by two-way ANOVA, followed by Tukey’s post hoc test (a P value < 0.05 was considered significant). Unlike wild type counterparts, both mutants did not resist a third increment of frequency. For this reason, panel C presents three increments of frequency over the basal heart rate and panels d and e shows two increments of frequency. CONTROL: Heated: N = 20, 19, 13, 5. Not heated: N = 20, 17, 14, 4. SERCA^A617T^: Heated: N = 8, 10, 8. Not heated: N = 9, 9, 3. SERCA^E442K^: Heated: N = 6, 5, 4. Not heated: N = 6, 5, 3 at basal, ΔFr1, ΔFr2, and ΔFr3 (only for CONTROL) respectively.

Heterozygous mutants and control animals were exposed to increasing stimulation frequencies and afterwards, their cardiac activity was recorded. Supplementary Figure S4 shows that basal spontaneous heart rates of wild type and conditional SERCA mutants did not significantly differ among them. The mutant phenotypes were induced by heat shock. Unlike control animals, neither mutant endured more than two frequency increments. SERCA^A617T^ exhibited a positive staircase, which was not observed in the control individuals. By contrast, SERCA^E442K^ showed a considerably more accented negative staircase than control individuals (Fig. 2a). The effects of the individual mutations are rather strong, considering that each line still holds one wild-type copy of SERCA. Temperature did not change the pattern of the staircase in control flies, but showed a clear opposite staircase in untreated mutant individuals that appeared to be different to control flies. This indicated that the presence of each mutation *per se*, even without the phenotype being induced by heat shock, modified cardiac activity (Fig. 2c,d). In contrast, the SERCA mutations did no modify half time of relaxation (Fig. 2b) in relation to basal frequency, all along the stimulation protocol. These alterations in the staircase pattern cannot be explained by changes in basal SR Ca^2+^ load. Supplementary Figure S5 shows that whereas the heat shock produces a significant increase in SR Ca^2+^ content in control flies, no significant changes were observed in mutant flies, either with or without heat shock.

### SERCA^A617T^ mutation is associated with enhanced arrhythmia and heart rate variability

**Figure 3:**
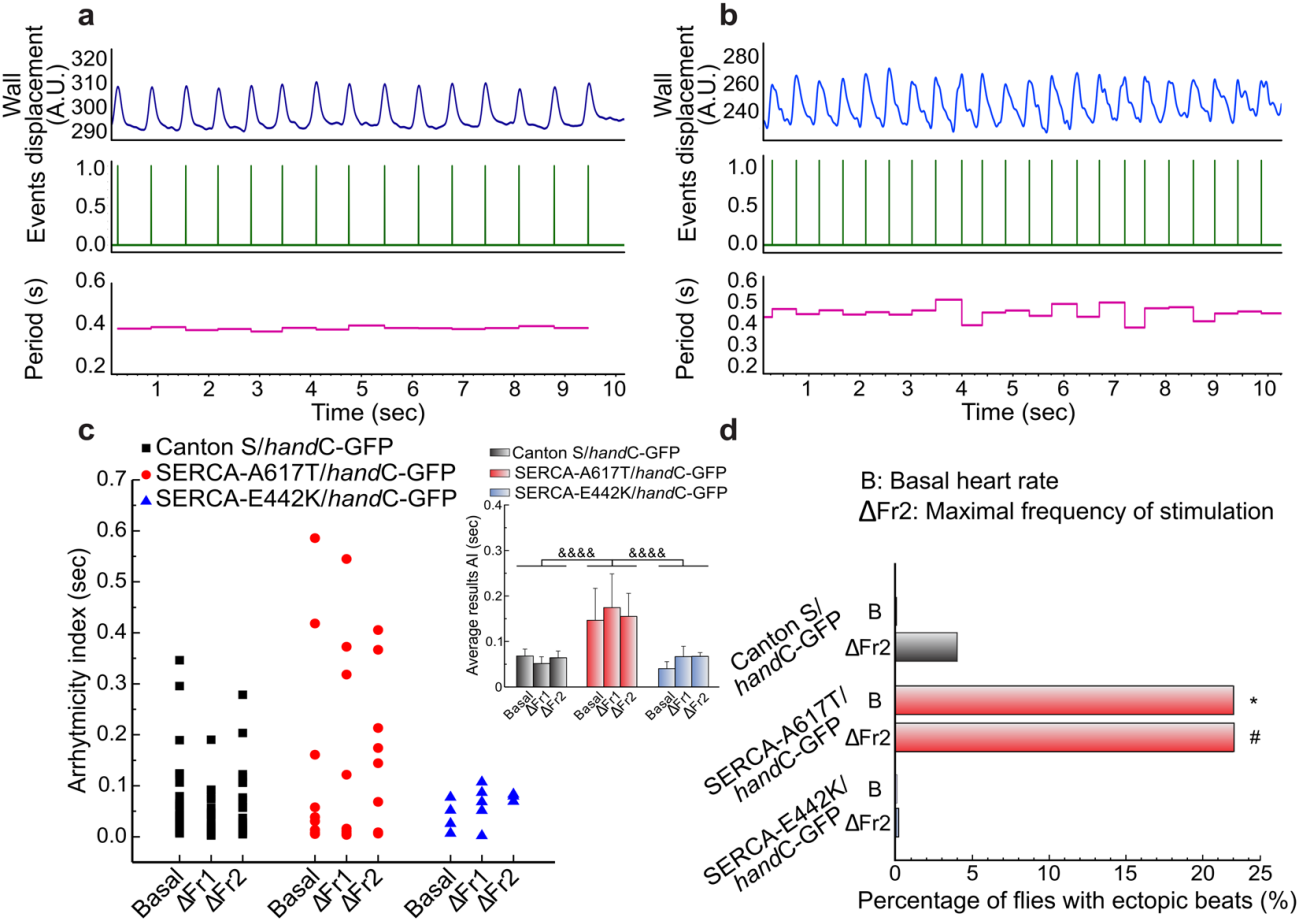
The SERCA^A617T^ mutation induces arrhythmia. (**a**,**b**) Typical recordings comparing two individuals with regular and irregular heart rate (**a**,**b**). Top: wall displacement, middle panel: each line indicates one event ( = one beat), bottom: periods (intervals of time between maximum of two consecutive peaks) are more variable in (**b**,**c**) Raw data and average results (inset) of arrhythmicity index. Mutant SERCA^A617T^ animals exhibit significantly increased heart rate variability at basal heart rate and at the maximal frequency of stimulation. (**d)** Increased proportion of animal mutant for SERCA^A617T^ exhibit incremented ectopic beats at basal rates and at the maximal frequency of stimulation. Arrhythmia: Control: N = 29, 25, 2. SERCA^A617T^ N = 9, 8, 9. SERCA^E442K^ N = 4, 4, 3. Two way ANOVA followed by Tukey’s test for multiple comparisons was used for statistical analysis. *P < 0.05 (between strains only in basal stimulation), ^#^P < 0.05 (between strains at different frequency stimulation), & P < 0.05 (between strains).

We next explored the effects of increasing the stimulation frequency on heart rate variability and arrhythmia generation. To this purpose, we analyzed and compared the arrhythmicity index^21^ (that represent heart rate variability^17^) and the proportion of flies that exhibit ectopic beats at the different stimulation frequencies explored. Figure 3, shows typical recordings of individuals with a regular and irregular heart rate (Fig. 3a,b, respectively). Each recording is accompanied by a graphical representation of periods (time between maximums). As shown by the period representation, Fig. 3b depicts a major variability of periods than that showed in Fig. 3a. In control flies, the arrhythmicity index was not influenced by any of the frequencies explored (Fig. 3c) and the higher stimulation frequency produced only a minor increase in the percentage of flies with ectopic beats (Fig. 3d). By contrast, the SERCA^A617T^ mutation caused a significant increase in ectopic beats and in the arrhythmicity index, both, at the basal level and at the highest stimulation frequency explored (Fig. 3c,d). Interestingly, the SERCA^E442K^ mutation did not affect the arrhythmicity index and did not induce ectopic beats (Fig. 3c,d). Previous studies by Sanyal *et al*.^8^ described that the SERCA^E442K^ mutant displays a decrease in basal heart frequency and an increase in arrhythmicity^9^. The reason for this discrepancy with the present results cannot be finally ascertained from our experiments. However, a possible clue to explain these discrepant results may be found in the different developmental stages studied (adult flies in our case and pupae in Sanyal *et al*. experiments) or the different preparations used (semi intact preparation vs. intact specimens). In contrast, our results agreed with data obtained from intact flies expressing SERCA^E442K^ in cardiac tissue, which after heat shock did also not exhibit any significant change in heart rate^20^.

## Discussion

The present study demonstrated that *D*. *melanogaster* shows a negative staircase. Possibly more important, our results further showed that distinct point mutations in SERCA directly affect the staircase pattern. Finally, the SERCA^A617T^ mutation is associated with an enhanced propensity to arrhythmia. Thus, our data reveal a direct contribution of SERCA to the intrinsic mechanisms of heart regulation and emphasize the importance that a putative dysfunction may have on heart performance.

Although both SERCA mutations tested are described to cause disrupted SERCA, they produced different effects at the molecular level: Replacement of alanine by threonine in the SERCA^A617T^ mutant converts a hydrophobic amino acid into a potentially phosphorylatable one, whereas replacement of glutamic acid for lysine in SERCA^E442K^ induces a significant alteration of the amino acid charge. Moreover, each mutation has a highly distinct impact on heart physiology. Whereas SERCA^A617T^ produced a positive staircase, SERCA^E442K^ accentuated the negative staircase observed CONTROL flies. Interestingly, both mutations *per se* produced a staircase pattern opposite to the one observed after heat shock activation of the phenotype.

The mechanisms by which these mutations alter the staircase phenomenon remain unclear yet. However, previous experiments demonstrated that the negative staircase effect observed in rat and mouse models is at least in part explained by the rather high SR Ca^2+^ content of both species under basal conditions^13^. This seems not to be the case in fly hearts. No significant differences were found in basal SR Ca^2+^ contents in the different groups of flies, but in control flies after the heat shock. However control flies presented a similar negative staircase under both conditions. The cause of the increase in the SR Ca^2+^ content in control flies is not clear from these experiments but might be a consequence of mechanisms activated by heat shock that modify the activity of SERCA. These mechanisms would be counteracted in both heterozygous mutants by the altered SERCA molecules. Taken together these results strongly suggest that the basal SR Ca^2+^ content does not play a significant role in the staircase pattern of the fly. Of note, corresponding similarities in Ca^2+^ content have already been reported in neural tissue and cultured cells tissue^8,10^. The lack of changes in the basal RS Ca^2+^ content does not exclude the possibility of changes in the SR Ca^2+^ content during the positive and negative staircase observed in SERCA^A617T^ and SERCA^E442K^ respectively. In rat ventricular myocytes the negative staircase in intracellular Ca^2+^ transients was associated to a similar beat-dependent decrease in the duration of the action potential, manifested primarily as a gradual loss of the action potential plateau^22^. The negative staircase of human heart failure is furthermore associated to a decrease in L-type Ca^2+^ current^23^. Interestingly, gated voltage Ca^2+^ current measured in larval skeletal muscle in SERCA^E442K^ mutants after heat shock, are significantly reduced^8^. A similar reduced Ca^2+^ entry due to action potential shortening in SERCA^E442K^ mutants hearts might have contributed to the exacerbated negative staircase exhibited by these flies eventually decreasing the Ca^2+^ availability in the cytosol. The mutation *per se*, without heat shock, manifests the opposite pattern, emphasizing the functional importance of the putative conformational change induces by heat shock. Regarding the SERCA^A617T^ mutation and to the best of our knowledge, there are not previous reports about a possible link between mutation and ionic currents. This might suggest that this amino acidic change does not modify the Ca^2+^ current in the same way as the SERCA^E442K^ mutation does. SERCA is a pleiotropic gene with several phenotypes, i.e. paralysis and incoordination and a detailed mechanistic analysis of the independent point mutations is required for a clearer understanding of fly cardiac responses to an increment in heart rate. While such analyses are beyond the scope of this work, our data emphasize the importance of SERCA function to the response of the fly to heart rate increases.

The results further indicate that ectopic beats as well as the arrhythmicity index are exacerbated in SERCA^A617T^ mutants. The origin of this arrhythmic pattern is not apparent to us. Since our experiments were performed in decapitated individuals, the influence of the autonomic nervous system in these alterations seems rather unlikely. Recent models^24^ emphasize the presence of a tight interaction between Ca^2+^ cycling proteins, including SERCA and cardiac automaticity, making SERCA mutations good candidates to explain the alterations of cardiac rhythm described here.

In conclusion, the results presented here demonstrate that *D*. *melanogaster* shows a negative staircase and that distinct point mutations in SERCA directly affect the type of staircase occurring within the heart. Our data further reveal a direct contribution of SERCA to the intrinsic mechanisms of heart regulation and emphasize the physiological importance of a putative dysfunction. Overall, this work establishes a molecular link for understanding a key physiological event in cardiac function and provides a new model for further research in vertebrates and humans.

## Materials and Methods

### *Drosophila* stocks, rearing and crosses

Fly stocks were amplified and maintained in vials at 28 °C, partially filled with a mixture of maize flour, glucose, agar and yeast supplemented with 10% antimycotic.

Assays were made with different strains obtained from Bloomington *Drosophila* Stock Center. Canton-S (BDSC# 9514, Sharma *et al*.^25^), is a control line. The other two strains were conditional mutants for the *Drosophila* SERCA gene (dSERCA, Ca-P60A). The first strain, express the mutant protein SERCA [E444K]. This conditional mutant, named Ca-P60A[Kum170], (BDSC # 26700)^8,9^ holds the amino acid replacement E442K of the protein encoded by the Ca-P60A gene. Activation of this dominant mutation induces paralytic phenotype and is also recessively lethal. The second strain holds the protein SERCA [A617T] (BDSC # 58974)^10^, that is a conditional mutant strain carrying the amino acid change A617T of the protein encoded by the Ca-P60A gene. Mutation activation induces a *Drosophila* incoordination phenotype and is recessively lethal.

Flies harboring the reporter system GCaMP3 specifically expressed in heart cells were capable of sensing increments of cytosolic Ca^2+^ (UAS-GCaMP3/UAS-GCaMP3; *tin*CΔ4-Gal4,UAS-GCaMP3/ *tin*CΔ4-Gal4,UAS-GCaMP3). These flies were a gift from Dr. Mathew Wolf, from the University of Virginia, USA^12^. The *hand*C-GFP line, expressing the reporter protein GFP in heart and pericardial cells was provided by Prof. Achim Paululat (University of Osnabrück, Germany)^11^.

Control flies were crossed to individuals carrying one of these reporters. We analyzed heterozygous individuals of F1 that possess one copy of the reporter system in order to obtain information on mechanical activity (using the *hand*C-GFP reporter) or Ca^2+^ transients (using the UAS-GCaMP3 reporter). Mutant flies were crossed with *hand*C*-*GFP reporter and mechanical activity was evaluated.

### Activation of mutation

Heat shock was applied for 20 or 30 min either at 37 °C or 41 °C to activate SERCA^A617T^ and SERCA^E444K^ mutations, respectively. The heat shock induced muscle incoordination or paralysis phenotypes due to the individual mutations present in the protein^8–10^.

### Evaluation of cardiac function in semi intact preparations

The dissection of adult hearts was performed as described Santalla *et al*.^17^. The procedure was performed on a Schonfeld Optik model XTD 217 stereomicroscope. Individuals between 7 and 11 days old were briefly anesthetized with carbon dioxide (CO_2_), and placed in a 60 mm Petri dish containing Vaseline and fixed by the dorsal region. The head and thorax were removed in this preparation; therefore neuronal influence on the cardiac activity was not significant. The middle ventral region of the abdomen was opened and the internal organs were removed. The preparation was submerged in oxygenated artificial hemolymph solution containing 5 mM KCl, 8 mM MgCl_2_, 2 mM CaCl_2_, 108 mM NaCl, 1 mM NaH_2_PO_4_, 5 mM HEPES,4 mM NaHCO_3_, 10 mM trehalose, 10 mM sucrose, pH 7.1^26^. Calcium recordings and mechanical activity of these semi intact preparations were carried out using a Carl Zeiss LSM410 and LSM800 confocal microscopes.

### Mechanical activity and calcium transient recording

Mechanical activity was recorded from all flies harboring the GFP protein under control of the *hand*C driver, expressed in cardiomyocytes and pericardial cells. A pericardial cell or a cardiomyocyte was focused with a 100X objective. We then tracked the fluorescent signal of a cardiac or pericardial cell edge at one of the two sides of the heart. The movement of a single cell expressing the GFP reporter was followed with laser scanning by setting a line along the displacement of either a pericardial cell or a cardiomyocyte. Recordings were obtained during 10 sec (Supplementary Video 1) intervals.

### Generation and adaptation of software for data analysis

The images obtained were processed with an algorithm for Anaconda developed in our laboratory to obtain sequentially in time the intensity of fluorescence of a pericardial/cardiac cell over a threshold value, and convert it in a digitalized image of cell displacement. A similar procedure was carried out to obtain a digitalized recording from Ca^2+^ transient images. All functional parameters were measured after verification that each recording had followed all stimulation frequencies tested. Values expressed as change of fluorescence along time, were analyzed with LabChart software (AD Instruments, CO, USA).

We measured fluorescence intensity (F-F_0_/F_0_) and t_1/2_, defined as the time from maximum fluorescence until half of the relaxation. Heart period was defined as the interval between two consecutive peaks. Arrhythmicity index (AI) was calculated as the standard deviation of periods normalized by their average. Additionally, we counted ectopic beats, which were defined as events that occurred immediately after the maximum was reached and before the finalization of a relaxation phase of a beat.

Ca^2+^ transients were registered by the fluorescence produced by Ca^2+^ binding to the GCaMP3 reporter. Semi intact preparations were visualized using a 5X objective and the laser was focused to stimulate a minimal central region of the conical chamber where the intensity of signal was higher than in other regions of the preparation. Recordings of 60 sec allowed us to obtain a measurable pattern of Ca^2+^ transients.

### Stimulation protocol

The semi intact preparation immersed in artificial hemolymph was electrically stimulated using a conducting device connected to an electro-stimulator and computer. Neutral pH was essential to complete the sequence of stimulation, in agreement with previous studies^27^. A customized chamber was designed to provide electrical field stimulation. Electrical pulses of 16 msec duration were applied. The intensity of the pulses varied to reach the excitability threshold at all the frequencies analyzed. The range of frequencies assayed was 1–6 Hz over the basal spontaneous frequency and the stimulation frequency was set according to the basal heart rate. From this basal rate, stimulation frequency was increased by 0.5 Hz steps. Supplementary Figure S6 schematizes the pacing protocol.

To estimate the sarcoplasmic reticulum Ca^2+^ load in basal conditions, a pulse of 10 mM of caffeine was applied. This compound produces the release of calcium content through the RyR2. The amplitude of the fluorescence induced by massive calcium release was calculated for all compared fly lines with and without heat shock.

### Statistical analysis

One way ANOVA andTwo way ANOVA followed by Tukey’s post hoc test, was utilized to compare differences among groups. Categorical data were expressed as percentages and compared with Fisher’s exact test. A p value < 0.05 was considered significant.

## Electronic supplementary material

Supplementary information

Supplementary video 1
