# Supplementary material for: SERCA is critical to control the Bowditch effect in the heart
Source: Sci Rep. 2018 Aug 20;8:12447. doi: 10.1038/s41598-018-30638-9 (PMC6102201; doi:10.1038/s41598-018-30638-9)
Supplement: Supplementary file 1 — Supplementary information [file 41598_2018_30638_MOESM1_ESM.pdf]

SERCA is critical to control the Bowditch effect in the heart

Darío Balcazar, Victoria Regge, Manuela Santalla, Heiko Meyer, Achim Paululat, Alicia Mattiazzi, Paola Ferrero

Supplementary Information

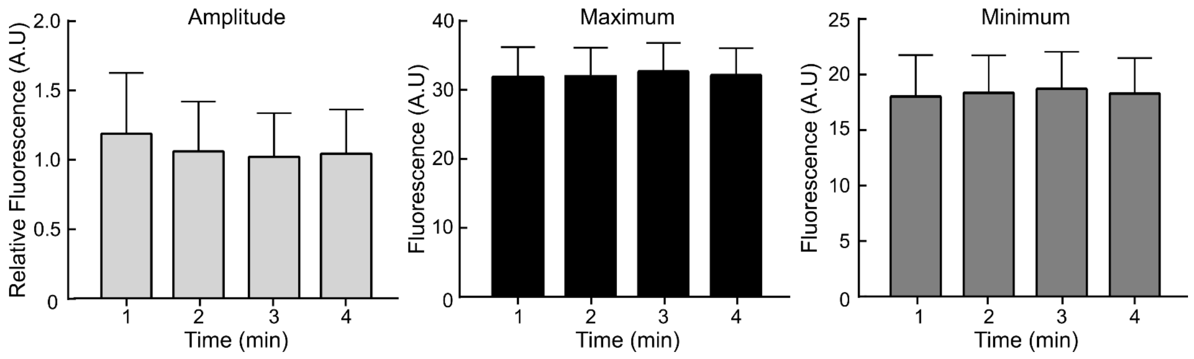

Supplementary Figure S1. The fluorescent signal of the GCaMP3 sensor remains unchanged with time in the absence of electrical stimulation.

GCaMP3 fluorescence was measured in flies' functional hearts during one minute intervals (total duration: 4 min). The panels correspond to the mean value of maxima, minima and the difference between both (amplitude) of fluorescent signals obtained at different intervals, respectively. Data represent mean values ± S.E., and significance was evaluated by one-way ANOVA, followed by Tukey's post hoc test. N = 7.

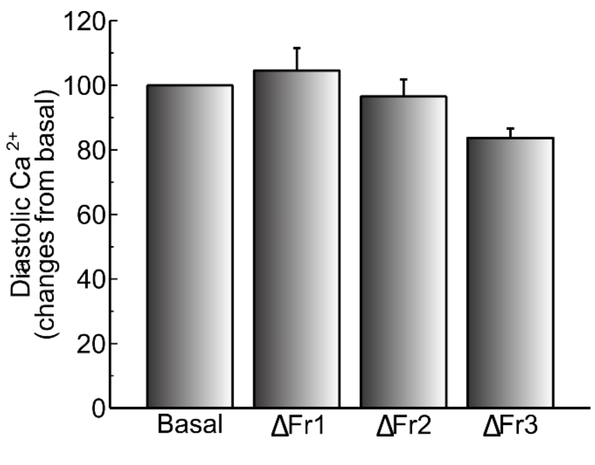

Supplementary Figure S2. Diastolic calcium does not change with incrementing the frequency of stimulation.

Average results shows that diastolic calcium does not significantly change with the increment of frequency. The results are visualized as percentage of change related to the basal cardiac frequency. Data represent mean values ± S.E., and significance was calculated by one-way ANOVA, followed by Tukey’s post hoc test. N = 11, 11, 7, 3 at basal, ΔFr1, ΔFr2, and ΔFr3 respectively.

```
sp|P16615|AT2A2_HUMAN              MENAHTKTVEEVLGHFGVNESTGLSLEQVKKLKERWGSNELPAEEGKTLLELVIEQFEDL
tr|A0A0B4LGB7|A0A0B4LGB7_DROME     MEDGHSKTVEQSLNFFGTDPERGLTLDQIKANQKKYGPNELPTEEGKSIWQLVLEQFDDL
                                   **:.*:****: *..**.: . **:*:*:*  ::.:*.****:****:: :**:***:**

sp|P16615|AT2A2_HUMAN              LVRILLLAACISFVLAWFEEGEETITAFVEPFVILLILVANAIVGVWQERNAENAIEALK
tr|A0A0B4LGB7|A0A0B4LGB7_DROME     LVKILLLAAIISFVLALFEEHEETFTAFVEPLVILLILIANAVVGVWQERNAESAIEALK
                                   **.****** ****** *** ***:******:******:***:**********.******

sp|P16615|AT2A2_HUMAN              EYEPEMGKVYRQDRKSVQRIKAKDIVPGDIVEIAVGDKVPADIRLTSIKSTTLRVDQSIL
tr|A0A0B4LGB7|A0A0B4LGB7_DROME     EYEPEMGKVVRQDKSGIQKVRAKEIVPGDLVEVSVGDKIPADIRITHIYSTTLRIDQSIL
                                   ********* ***...:*.:.**:*****:**::****:*****:* * *****:*****

sp|P16615|AT2A2_HUMAN              TGESVSVIKHTDPVPDPRAVNQDKKNMLFSGTNIAAGKAMGVVVATGVNTEIGKIRDEMV
tr|A0A0B4LGB7|A0A0B4LGB7_DROME     TGESVSVIKHTDAIPDPRAVNQDKKNILFSGTNVAAGKARGVVIGTGLSTAIGKIRTEMS
                                   ************.:************:******:***** ***:.**:.* ***** **

sp|P16615|AT2A2_HUMAN              ATEQERTPLQQKLDEFGEQLSKVISLICIAVWIINIGHFNDPVHGGSWIRGAIYYFKIAV
tr|A0A0B4LGB7|A0A0B4LGB7_DROME     ETEEIKTPLQQKLDEFGEQLSKVISVICVAVWAINIGHFNDPAHGGSWIKGAIYYFKIAV
                                    **: .******************:**:*** *********.******.**********

sp|P16615|AT2A2_HUMAN              ALAVAAIPEGLPAVITTCLALGTRRMAKKNAIVRSLPSVETLGCTSVICSDKTGTLTTNQ
tr|A0A0B4LGB7|A0A0B4LGB7_DROME     ALAVAAIPEGLPAVITTCLALGTRRMAKKNAIVRSLPSVETLGCTSVICSDKTGTLTTNQ
                                   ************************************************************

sp|P16615|AT2A2_HUMAN              MSVCRMFILDRVEGDTCSLNEFTITGSTYAPIGEVHKDDKPVNCHQYDGLVELATICALC
tr|A0A0B4LGB7|A0A0B4LGB7_DROME     MSVSRMFIFDKVEGNDSSFLEFEMTGSTYEPIGEVFLNGQRIKAADYDTLQELSTICIMC
                                   ***.****:*.***: .*: ** :***** *****. :.: ::. :** * **:*** :*
                                                         ⬇E442K
sp|P16615|AT2A2_HUMAN              NDSALDYNEAKGVYEKVGEATETALTCLVEKMNVFDTELKGLSKIERANACNSVIKQLMK
tr|A0A0B4LGB7|A0A0B4LGB7_DROME     NDSAIDYNEFKQAFEKVGEATETALIVLAEKLNSFSVNKSGLDRRSAAIACRGEIETKWK
                                   ****:**** *  .:***********  *.**:* *..: .**.. . * **.. *:   *

sp|P16615|AT2A2_HUMAN              KEFTLEFSRDRKSMSVYCTPNKPSRTSM-SKMFVKGAPEGVIDRCTHIRVGSTKVPMTSG
tr|A0A0B4LGB7|A0A0B4LGB7_DROME     KEFTLEFSRDRKSMSSYCTPLKASRLGTGPKLFVKGAPEGVLERCTHARVGTTKVPLTSA
                                   *************** **** *.**  .  .*:*********::**** ***:****:**.

sp|P16615|AT2A2_HUMAN              VKQKIMSVIREWGSGSDTLRCLALATHDNPLRREEMHLEDSANFIKYETNLTFVGCVGML
tr|A0A0B4LGB7|A0A0B4LGB7_DROME     LKAKILALTGQYGTGRDTLRCLALAVADSPMKPDEMDLGDSTKFYQYEVNLTFVGVVGML
                                   :* **:::  ::*:* *********. *.*:. :** * **::* :**.****** ****
                                             ⬇A617T
sp|P16615|AT2A2_HUMAN              DPPRIEVASSVKLCRQAGIRVIMITGDNKGTAVAICRRIGIFGQDEDVTSKAFTGREFDE
tr|A0A0B4LGB7|A0A0B4LGB7_DROME     DPPRKEVFDSIVRCRAAGIRVIVITGDNKATAEAICRRIGVFAEDEDTTGKSYSGREFDD
                                   **** ** .*:  ** ******:******.** *******:*.:***.*.*::*****:

sp|P16615|AT2A2_HUMAN              LNPSAQRDACLNARCFARVEPSHKSKIVEFLQSFDEITAMTGDGVNDAPALKKAEIGIAM
tr|A0A0B4LGB7|A0A0B4LGB7_DROME     LSPTEQKAAVARSRLFSRVEPQHKSKIVEFLQSMNEISAMTGDGVNDAPALKKAEIGIAM
                                   *.*: *. *   .:* *:****.***********::**:*********************

sp|P16615|AT2A2_HUMAN              GSGTAVAKTASEMVLADDNFSTIVAAVEEGRAIYNNMKQFIRYLISSNVGEVVCIFLTAA
tr|A0A0B4LGB7|A0A0B4LGB7_DROME     GSGTAVAKSAAEMVLADDNFSSIVSAVEEGRAIYNNMKQFIRYLISSNIGEVVSIFLTAA
                                   ********:*:**********:**:***********************:****.******

sp|P16615|AT2A2_HUMAN              LGFPEALIPVQLLWVNLVTDGLPATALGFNPPDLDIMNKPPRNPKEPLISGWLFFRYLAI
tr|A0A0B4LGB7|A0A0B4LGB7_DROME     LGLPEALIPVQLLWVNLVTDGLPATALGFNPPDLDIMEKPPRKADEGLISGWLFFRYMAI
                                   **:**********************************:****:..* **********:**

sp|P16615|AT2A2_HUMAN              GCYVGAATVGAAAWWFIAADGGPRVSFYQLSHFLQCKEDNPDFEGVDCAIFESPYPMTMA
tr|A0A0B4LGB7|A0A0B4LGB7_DROME     GFYVGAATVGAAAWWFVFSDEGPKLSYWQLTHHLSCLGGGDEFKGVDCKIFSDPHAMTMA
                                   * **************: :* **.:*::**:*.*.*   .. :*:**** **..*:.****

sp|P16615|AT2A2_HUMAN              LSVLVTIEMCNALNSLSENQSLLRMPPWENIWLVGSICLSMSLHFLILYVEPLPLIFQIT
tr|A0A0B4LGB7|A0A0B4LGB7_DROME     LSVLVTIEMLNAMNSLSENQSLITMPPWCNLWLIGSMALSFTLHFVILYVDVLSTVFQVT
                                   ********* **:*********: **** *:**:**:.**::***:****: *. :**:*

sp|P16615|AT2A2_HUMAN              PLNVTQWLMVLKISLPVILMDETLKFVARNYLEPGKECVQPATKSCSFSACTDGISWPFV
tr|A0A0B4LGB7|A0A0B4LGB7_DROME     PLSAEEWITVMKFSIPVVLLDETLKFVARK-------------------------------
                                   **.. :*: *:*:*:**:*:*********:

sp|P16615|AT2A2_HUMAN              LLIMPLVIWVYSTDTNFSDMFWS
tr|A0A0B4LGB7|A0A0B4LGB7_DROME     ----------IADVNPRFH-----
                                             : ..:..*
```

11

Supplementary Figure S3. Pairwise alignment of human (P16615_AT2A2) and *D. melanogaster* (A0A0B4LGB7_DROME) SERCA.

Point mutations in *D. melanogaster*' SERCA are indicated by blue or red arrow for E442K and A617T, respectively.

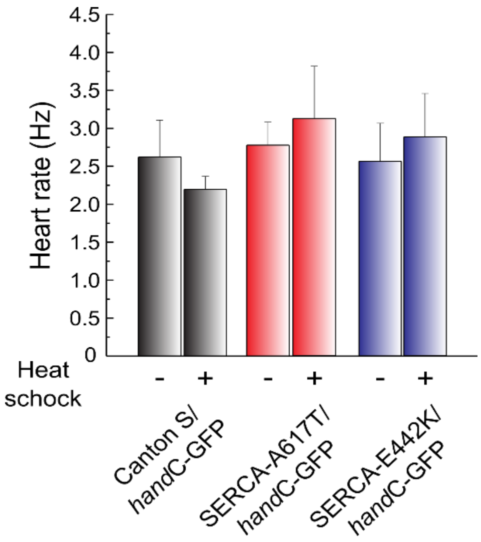

Supplementary Figure S4. Spontaneous heart rates of control flies and conditional SERCA mutants are similar.

Spontaneous heart rates (Hz) of control flies (CantonS/*hand*C-GFP, black bars) and of two strains harboring individual conditional mutations in SERCA (SERCA^{A617T}/*hand*C-GFP or SERCA^{E442K}/*hand*C-GFP, red and blue bars, respectively) were determined in individuals pre-treated (+) or not (-) by heat shock. Data represent mean values ± S.E.; significance was calculated by two-way ANOVA, followed by Tukey's post hoc test. N= 16, 14, 8 (not heated) and 7, 13, 10 (heated) for WT, SERCA^{A617T} and SERCA^{E442K} respectively.

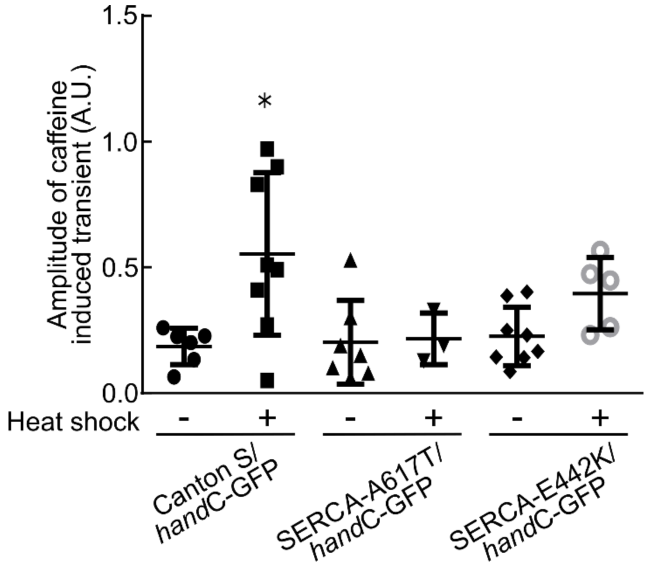

Supplementary Figure S5. Sarcoplasmic reticulum calcium load does not change in mutants flies.

SR calcium load was estimated by applying a pulse of caffeine (10 mM) to semi-intact preparation. Figure shows individual and average values of the caffeine induced peak amplitude. Only heat shocked control flies exhibit an increment in calcium content whitin the SR. N = 7. Significance was calculated by two-way ANOVA, followed by Tukey's post hoc test (* $P < 0.05$). N= 6, 6, 8 (not heated) and 8, 3, ,5 (heated) for WT, SERCA^{A617T} and SERCA^{E442K} respectively.

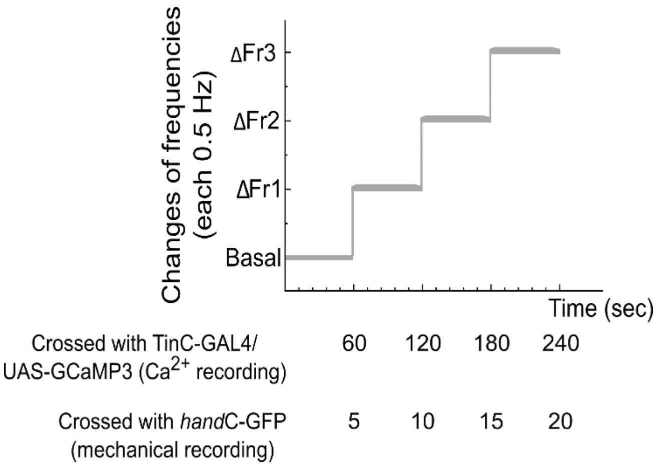

Supplementary Figure S6. Graphical representation of electrical stimulation changes induced in the *D. melanogaster* heart.

D. melanogaster hearts were electrically paced at different frequencies by applying discrete changes of 0.5 Hz. Corresponding changes were implemented in 60 sec intervals or in 5 sec intervals, for Ca^{2+} or mechanical recording, respectively.

Supplementary Video S1. Heartbeat of *Drosophila melanogaster*.

Fluorescence of cardiomyocytes and pericardial cells harboring the reporter system *hand*C-GFP allows following the movement of the heart walls. Mechanical activity of control, $SERCA^{A617T}$, and $SERCA^{E442K}$ mutant animals was determined by tracking lateral movement of cells.
